# The dark side of circulating nucleic acids

**DOI:** 10.1111/acel.12454

**Published:** 2016-02-22

**Authors:** Silvia Gravina, John M. Sedivy, Jan Vijg

**Affiliations:** ^1^ Department of GeneticsAlbert Einstein College of MedicineBronxNY10461USA; ^2^ Department of Molecular Biology, Cell Biology and BiochemistryBrown UniversityProvidenceRI02912USA; ^3^ Present address: Illumina, Inc.San DiegoCA92122

**Keywords:** aging, cell‐free DNA, DNA damage, mutagenesis

## Abstract

Free circulating or cell‐free DNA (cfDNA), possibly from dying cells that release their contents into the blood as they break down, have become of major interest as a source for noninvasive diagnostics. Recent work demonstrated the uptake of human cfDNA in mouse cells *in vitro* and *in vivo*, accompanied by the activation of a cellular DNA damage response (DDR) and the appearance of apoptotic proteins in the host cells. By acting as a source of mobile genetic elements, cfDNA could be a continuous source of DNA mutagenesis of healthy cells in the body throughout life, promoting progressive cellular aging *in vivo*. As such, cfDNA may causally contribute to multiple aging‐related diseases, such as cancer, diabetes, and Alzheimer's disease.

Billions of cells in the adult human body are eliminated daily through cell death processes, such as apoptosis and necrosis; especially necrotic cells, which unlike apoptotic cells are not generally removed cleanly by phagocytosis, are thought to be a source of degraded DNA fragments released to the blood plasma or serum as cell‐free DNA or circulating free DNA (cfDNA) (van der Vaart & Pretorius, 2007). Noninvasive detection of cfDNA has the potential to advance new prognostic paradigms and to impact clinical protocols and treatment regimens in human disease (Cai *et al*., 2015). Indeed, cfDNA has the potential of becoming part of the standard of care in oncology, prenatal testing, transplant medicine, and cardiovascular disease (Sayres & Cho, 2011; Dinakaran *et al*., 2014; Cai *et al*., 2015) and is emerging as one of the most promising and exciting areas of medicine. It has already made a major impact on prenatal care, where fetal cfDNA in the circulation is now used for early diagnosis of genetic abnormalities, fetal sex, or pre‐eclampsia (Sayres & Cho, 2011; Bianchi *et al*., 2014). In oncology, while not clinically implemented yet, cfDNA can be used to probe cancer genome dynamics by analyzing plasma samples (Schwarzenbach *et al*., 2011). For example, when a biopsy is unavailable, cfDNA could be used as a ‘liquid biopsy’ to assess sensitivity and resistance to targeted therapies. In principle, cfDNA offers the opportunity to study cancer progression noninvasively by genome‐wide analysis of cfDNA in plasma.

While cfDNA‐based clinical applications continue to gain momentum, some aspects of the biology of cfDNA are still unexplored and several key questions remain. One question with high relevance to aging is whether or not cfDNA fragments can behave as mobile genetic elements, illegitimately integrating in the chromosomal DNA of healthy cells in its own host, thereby contributing to genome instability and possibly causing age‐related functional decline and age‐related pathophysiological processes. The first evidence that genomic DNA can be taken up by cultured cells was provided by Gartler (1959). These and later experiments were carried out using tritiated thymidine as a marker, with no direct evidence that the endogenous, undegraded DNA had integrated in the host genome. However, more recently, it was demonstrated that NIH‐3T3 cells incubated with plasma from patients with colon cancer were oncogenically transformed, strongly suggesting that the endogenous DNA in the plasma was taken up and integrated by the host cells (Garcia‐Olmo *et al*., 2010). Evidence has also been provided for the uptake and integration of Y‐chromosome‐specific DYS14 gene fragments in autopsied brain of a woman bearing a male fetus, suggesting fetal DNA in the bloodstream as its source (Chan *et al*., 2012).

Mittra *et al*., in a recent paper in the Journal of Biosciences (Mittra *et al*., 2015), demonstrated for the first time that cfDNA from cancer patients or control individuals are taken up by mouse 3T3 cells and localized within the nucleus. Chromatin (Cfs) appeared to be significantly more effective in this respect, which is in keeping with data showing that chromatin reconstituted with histone H2B proteins is more efficient than naked DNA in gene delivery to the nucleus of intact living mammalian cells, possibly due to DNA condensation and protection against degradation in the reconstituted chromatin (Wagstaff *et al*., 2008). Fluorescently labeled human cfDNA and Cfs isolated by Mittra *et al*. localized in the nuclei of the mouse cells within minutes, with almost 100% of the nuclei containing fluorescent signals within 30 min. FISH analysis as well as whole‐genome sequencing of single‐cell clones – derived from the NIH3T3 cells treated with human cfDNA and Cfs – revealed the presence of tens of thousands of human sequencing reads in the mouse cell genomes, including human Alu repeat elements. Furthermore, the authors reported the induction of γ‐H2AX foci, which co‐localized with sites of integration of Cfs in NIH3T3 cells, as well as caspase‐3 activity and expression of ATM, p53, p21, GADD‐34, and DNA ligase IV, indicative of increased genome instability, apoptosis, and activation of the DNA damage response. Evidence was also provided that the integration of cell‐free nucleic acid with host cells occurs *in vivo* as well as *in vitro*. BALB/C mice were injected intravenously with cfDNA and Cfs and FISH analysis of heart, lung, liver, and brain of the mice sacrificed 7 days after injection revealed genomic localization of nucleic acids, again Cfs localizing more efficiently than cfDNA. Of note, genomic integration of Cfs in the mouse brain indicated that chromatin particles are able to cross the blood–brain barrier. Like with the 3T3 cells, the authors showed the induction of γ‐H2AX, caspase‐3 and activation of the DNA damage response.

Hence, this recent work provides us with the first direct evidence that cfDNA from the circulation can behave like mobile genetic elements and integrate randomly in the genome of healthy cells *in vitro* and *in vivo*. As such, it offers a fascinating new mechanism of age‐related mutagenesis, highlighting the fate and effects of free nucleic acids within our body (schematically depicted in Fig. 1). However, many questions remain.

**Figure 1:**
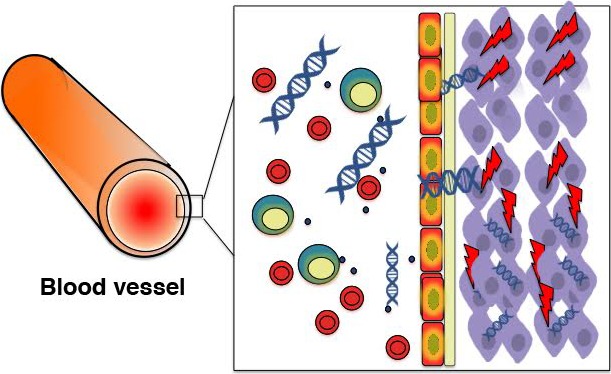
Free nucleic acids as a potential endogenous source of DNA damage. Upon degradation, possibly after cell death processes, cellular DNA is released into the peripheral bloodstream as cell‐free DNA (cfDNA). CfDNA is readily taken up by a variety of cells and integrated in the host genome. Integration in the host cell chromosomes results in the activation of a cellular DNA damage response (DDR).

Probably the most important uncertainty is the actual integration of the human cfDNA in the mouse genome. Mittra *et al*. reported human reads in sequence data obtained from their mouse samples, which does not, however, prove that the foreign DNA is not episomal or possibly a contaminant. Likewise, their observations that fluorescently tagged cfDNAs associate with chromosomes in mitosis and that the cfDNAs are stably propagated during cell division in culture are consistent with but do not directly show genomic integration. To further substantiate integration, it will be important to demonstrate the existence of actual junction fragments, that is, the juxtaposition of well‐annotated mouse with well‐annotated human sequences. This would not only provide definite evidence for integration, but also information as to the distribution of the integrated human cfDNA across the mouse genome.

Second, the efficiency of the cfDNA being taken up by the cells, that is, virtually 100% of all cells transfected in 30 min, is very high. This was also true for apoptosis: About 40% of cells were made apoptotic as a consequence of the treatment of cells with 5 ng of cfDNA. This is as potent as some high‐powered clastogens, such as etoposide or cisplatin. It is not entirely clear whether this high potency is predominantly associated with cfDNA from cancer patients, although this was apparently more efficient in inducing γgH2AX foci and the DNA damage response. In this respect, it would be important to test DNA from various sources, for example, bacterial, yeast, or other mammalian species, to establish the universality of the highly efficient uptake of the human DNA by mouse cells.

Third, when trafficking through the cell to integrate into the nucleus, how does the cfDNA evade the cytosolic DNA sensors of the innate immune system, expected to degrade foreign DNA through such pathways as the STING–TBK1–IRF3 axis?

Finally, probably the most interesting question is whether cfDNA truly behaves as mobile genetic elements under normal conditions. That is, rather than extracting concentrated cfDNA from heterogenic serum samples and intravenously injecting that in the mouse, integration of its own cfDNA should be studied, for example, as a function of age. Because integrated DNA fragments can then no longer be uniquely aligned as foreign DNA to a reference sequence, single cells or clones should be studied for insertion events as compared to the germline sequence, which is considerably more difficult than screening for reads containing human sequences.

Hence, while still lacking in important details, the work by Mittra *et al*. opens up the intriguing prospect of a new, endogenous source of genome instability that could well contribute to increased genome mosaicism with age (Vijg, 2014). In this respect, cfDNA could act similarly to the previously described age‐related derepression of endogenous retrotransposons in the somatic genome during aging (De Cecco *et al*., 2013). In this respect, there is evidence that cfDNA becomes increasingly frequent in the circulation as a function of age (Jylhava *et al*., 2011), for example, due to increased vulnerability of aged and damaged cells to cell death (Pollack & Leeuwenburgh, 2001). Its activation of the DNA damage response could increase the level of genome instability considerably, contributing to aging‐related degenerative processes, such as cellular senescence, cancer, and inflammation. Further research on the biological and pathological roles of cell‐free nucleic acids will help to elucidate its importance as an intrinsic mechanism of aging.
